# Common Resistance Patterns in the Burn Unit of a Tertiary Care Center: A Retrospective Observational Study

**DOI:** 10.7759/cureus.43896

**Published:** 2023-08-22

**Authors:** Mohammed S Alqarni, Meshari Attar, Salem Alshammari, Badr Ambon, Abdulrahman A Al Zhrani, Abdullah Alghamdi, Ahmad Naebulharam, Abdulfattah Al-Amri, Hadeel Altayib

**Affiliations:** 1 Internal Medicine, King Abdullah International Medical Research Center, Jeddah, SAU; 2 Internal Medicine, National Guard Hospital, King Abdulaziz Medical City, Jeddah, SAU; 3 Research Office, King Abdullah International Medical Research Center, Jeddah, SAU; 4 College of Medicine, King Saud Bin Abdulaziz University for Health Sciences, Jeddah, SAU; 5 Emergency, Ministry of Health, Mikhwah General Hospital, Al-Baha, SAU; 6 Critical Care Medicine, King Faisal Specialist Hospital & Research Center, Riyadh, SAU; 7 Radiology, National Guard Hospital, King Abdulaziz Medical City, Jeddah, SAU; 8 Radiology, King Abdullah International Medical Research Center, Jeddah, SAU; 9 Pathology and Laboratory Medicine, King Abdullah International Medical Research Center, Jeddah, SAU; 10 Pathology and Laboratory Medicine, National Guard Hospital, King Abdulaziz Medical City, Jeddah, SAU; 11 Internal Medicine, King Saud Bin Abdulaziz University for Health Sciences, Jeddah, SAU

**Keywords:** mortality, antibiotic resistance, antibiotic sensitivity, burn wound infection, burn

## Abstract

Background

Nosocomial bacterial infections have been one of the major concerns in the healthcare system. Burn patients, specifically severe cases, are at a high risk of developing bacterial infections compared to others. The most frequent cultures among burn patients are *Staphylococcus aureus*, *Pseudomonas aeruginosa*,and* Acinetobacter baumannii. *There is a scarcity of local data showing the most common infections in burn patients. This research aimed to determine the most common organisms that cause infections in burn unit patients and the antibiotic sensitivity and resistance patterns in King Abdulaziz Medical City (KAMC) in Jeddah.

Methodology

In this cross-sectional study, data were collected from patients’ files into a data collection sheet. All patients in the burn unit with a positive culture were included in the study using a convenient sampling technique from Best-Care, KAMC electronic medical records. Burn patients with negative culture results and patients who were admitted to the plastic surgery ward for reasons other than burns were excluded. For sample size calculation, convenience sampling of 109 patient medical charts, over the study period from June 2016 to November 2021, was selected for data extraction, analysis, and reporting.

Results

*Pseudomonas aeruginosa* was the leading cause of infection in burn patients comprising 33.9% of the cases. *Enterobacter cloacae* was the second most frequent cause of infection among burn patients (27.5%). *Klebsiella pneumoniae* was the third most frequent cause of infection (26.6%) while *Acinetobacter baumannii* was the fourth most frequent cause of infection in burn patients (22.9%).

Conclusions

Understanding the local epidemiology of bacterial infections will be crucial for the development of treatment guidelines designed to standardize initial antibiotic use, reduce hospital-acquired infections, and reduce drug resistance. More attention should be paid to gram-negative bacteria, specifically *Pseudomonas aeruginosa* and *Enterobacter Cloacae*.

## Introduction

In burn injuries of all types, invasive infection is the main cause of morbidity and death which is responsible for 51% of deaths among burn patients [1]. Sepsis is more common in burn unit patients compared to trauma patients because of the deterioration in the first line of defense, the epidermis. Burn infections can delay the wound healing process and lead to serious systemic complications. As such, burn patients are more susceptible to hospital-acquired infections [2]. Every year, healthcare providers in the United States admit approximately half a million people, about 40,000 of those are burn injuries, with 75% requiring specialized treatment at a specialized burn center [1,2]. Burn infection is defined as the existence of high concentrations (>105 organisms per gram of tissue) of bacteria on the surface of the burn wound [1]. The natural pathophysiology of burns on a large surface area, i.e., burns that cover more than 20% of the total body surface area, weaken the barrier function of the skin which is responsible for preventing the access of microorganisms, triggering a lengthened exposure to host immune response mediators that is not seen in other critical illnesses [1].

Nosocomial bacterial infections are one of the major concerns in the healthcare system. One of the most common hospital-associated infections in burn unit patients are epidermis infections (wounds), pneumonia, and urinary tract infections [3]. *Staphylococcus aureus* along with *Pseudomonas aeruginosa* are known to be the most common microorganisms that take over burns [4]. A major method to improve outcomes is to identify the organism as early as possible. Burn patients, especially severe cases, are at a higher risk of developing bacterial infections than other patients. Recently, new resistant pathogens have forced healthcare practitioners to search for alternative forms of treatment for burn patients [3]. Several studies have been conducted globally to identify the most frequently occurring multiple-drug-resistant organisms in specific burn units, such as *Staphylococcus aureus, Pseudomonas aeruginosa,* and *Acinetobacter baumannii* [5]. However, the major cause of gram-positive burn wound infections worldwide remains multidrug-resistant *Staphylococcus aureus* [3]. Gram-positive bacteria are generally considered to be the main cause of burn infections. The excessive use of broad-spectrum antibiotics in the treatment of sepsis leads to the generation ofmethicillin-resistant *Staphylococcus aureus *(MRSA) [6]. This antibiotic-resistant strain does not cause an infection in uninjured individuals but opportunistically causes an infection in burn patients due to their compromised immunity [7]. Other frequently detected gram-positive bacteria in burn patients are *Enterococcus faecalis *and *Enterococcus faecium* [4]. *Pseudomonas aeruginosa* infection is also considered a major cause of morbidity in burn patients [8]. Frequently detected gram-negative bacteria in burn patients are *Escherichia coli* and *Klebsiella *[2]. As there is a scarcity of local data on bacterial infections in burn unit patients, initial antibiotic use is not standardized.

This study aims to identify bacterial infections and their relationship with the type, site, and degree of burn. In addition, this study aims to evaluate the recovery time of bacterial infections in response to different antibiotics, as well as to determine the relationship between antibiotic resistance and hospital stay. This study will help focus on common bacterial organisms that cause infections in burn patients which will help in establishing a standardized protocol for the use of antibiotics in burn units in King Abdulaziz Medical City (KAMC) in Jeddah.

## Materials and methods

In this cross-sectional study, all patients in the burn unit with a positive culture were included through Best-Care (electronic medical records) at KAMC. Burn patients with negative culture results and those who were admitted to the plastic surgery ward for reasons other than burns were excluded. This study was approved by the Institutional Review Board (IRB) at King Abdullah International Medical Research Center (approval number: #IRBC/2680/21).

The study population comprised hospitalized burn patients admitted to KAMC (Burn Unit Center) from June 2016 to November 2021. The medical charts of in-patients were collected which included data on age, sex, degree of burn, affected site, area percentage, type of burn, type of antibiotics, admission date, type and sample date, organism, length of stay, and complication after infection and survival. Regarding data entry quality control, data were double-checked by the research team and re-evaluated.

Quantitative numerical data and qualitative categorical data were analyzed. The mean and standard deviation were used to analyze the quantitative numerical data. The median and range were used to analyze the qualitative categorical data. Data were recorded in Excel and analyzed as qualitative and quantitative variables. Descriptive statistics and percentages were used in this study. The outcomes of the research were bacterial infections and their relationship with the type, site, and degree of burn; the complications of the burn-related infection; and the relationship between antibiotic resistance and hospital stay and mortality. Two-tailed Student’s t-test was used to compare means, and the chi-square test was performed for comparing categorical variables. A p-value less than 0.05 was considered significant. All results were computed using SPSS version 23 (IBM Corp., Armonk, NY, USA).

## Results

Of the total sample size of 109, 68 (62.0%) of the patients were males, whereas 41 (37.0%) were females. The mean age of the study population was 26.35 ± 23.07 years. Emergency department admission accounted for 88 (80.7%) of the total admissions. In total, 35 (32.1%) patients were admitted with second-degree burns, followed by 17 (15.5%) with third-degree burns, and one (3.1%) accounting for both fourth- and fifth-degree burns. In 39 (35.7%) of the patients, the upper body was affected, and 39 (35.7%) patients had their lower bodies affected. Full-body burns were reported in 24 (22%) patients. A total of 98 (89.9%) patients underwent surgery. Scalded burns were reported to be the leading cause of burn in 43 (39.4%) cases, followed by flame burns with the second highest number of cases at 35 (32.1%). Electrical shock burns and contact burns accounted for seven (6.4%) and seven (6.4%) of the cases, respectively (Table 1). The area affected by burns had a mean of 21.13 ± 19.08%, and the hospital stay had a mean of 46.05 ± 43.22 days.

**Table 1:** Demographics and characteristics of our study population. ER=Emergency Room

Demographics		n	%
Year	2016	14	12.8
	2017	29	26.6
	2018	21	19.2
	2019	13	11.9
	2020	2	1.8
	2021	30	27.5
Type of admission	Emergency room	88	80.7
	Inpatient	4	3.5
	Outpatient	2	1.8
	Transfer	15	13.7
Gender	Male	68	62.3
	Female	41	37.6
Degree of burn	Second degree	35	32
	Third degree	17	15.5
	Fourth degree	1	3.1
	Fifth degree	1	3.1
Affected site	Upper body	39	35.7
	Lower body	39	35.7
	Full body	24	22
Surgery	Yes	98	89.9
	No	11	10.1
Type of burn	Flame burn	35	32.1
	Scalded burn	43	39.4
	Electrical shock	7	6.4
	Chemical burn	6	5.5
	Contact	7	6.4
	Friction	5	4.5
	Coal	2	1.8
	Candle	1	0.9
	Dry heat	1	0.9
Mortality		31	28

**Figure 1:**
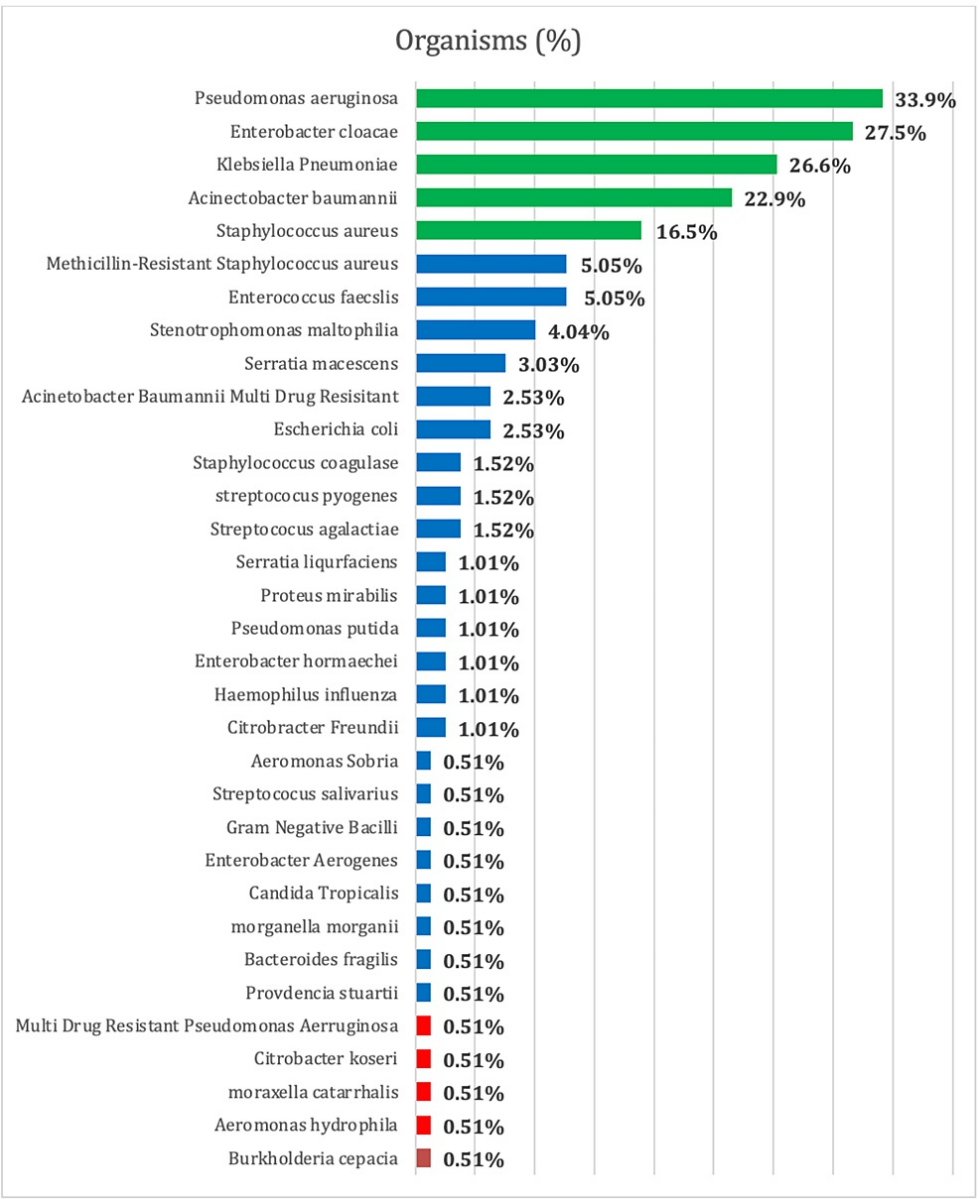
Type of organisms and their percentages.

*Pseudomonas aeruginosa* was the leading cause of infection in burn patients with the highest number of cases at 37 (33.9%), followed by *Enterobacter cloacae* in 30 (27.5%) cases, *Klebsiella pneumoniae* in 29 (26.6%) cases, *Acinetobacter baumannii* in 25 (22.9%) cases, and *Staphylococcus aureus* in 18 (16.5%) of the cases (Figure 1).

Burn patients with gram-negative bacterial infections required the longest duration of hospital stay, with an average of 41.35 days. Gram-positive bacterial infections had a hospital length of stay of 37.89 days. Burn patients infected with both gram-positive and gram-negative bacteria had an average length of stay at the hospital of 36.84 days. All types of bacterial infections were not associated with either prolonged hospital stay or mortality (p > 0.05).

Gram-negative bacteria were the leading causative organisms in infections involving upper-body burns, lower-body burns, as well as full-body burns. Both gram-positive and gram-negative bacteria were the second leading cause of infections across the three types of body burns. Gram-positive bacterial infections were the least reported in upper-body burns. Gram-negative bacteria combined with fungi were reported in only a single case of lower-body burn.

**Figure 2:**
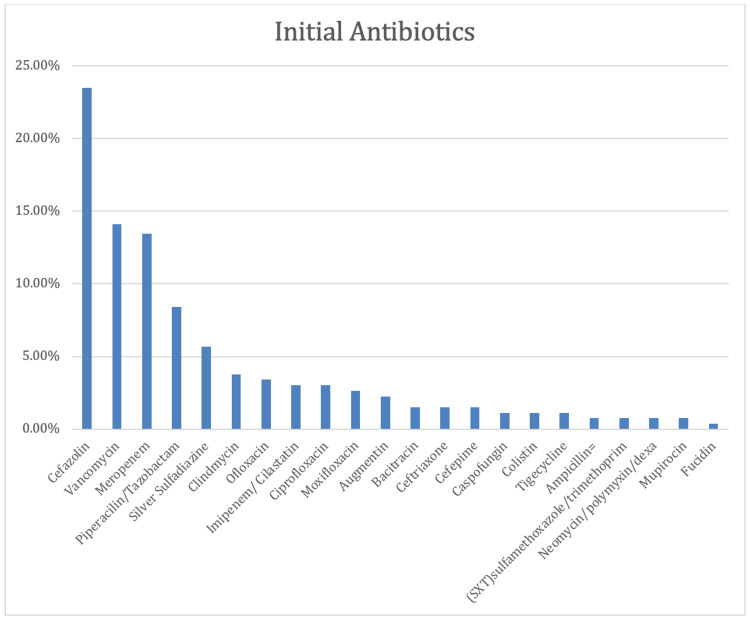
The initial antibiotic use.

Cefazolin was the most empirically used initial antibiotic in burn patients at 70 out of 297 times (23.5%). Vancomycin was used 42 times out of the total 297 (14.1%) as the second most empirically used initial antibiotic. Meropenem was the third most empirically used initial antibiotic at 40 out of 297 (13.46%), followed by piperacillin and tazobactam as the fourth most frequent initial antibiotics at 25 out of 297 (8.4%) (Figure 2).

The sensitivity and resistance patterns of the most commonly used antibiotics in these infections are outlined in Table 2.

**Table 2:** Antibiotic susceptibility of bacterial isolates. S = susceptible; R = resistant; - = not tested or not

Organism	CefazolinS/R	Piperacillin/Tazobactam S/R	Ciprofloxacin S/R	Ceftriaxone S/R	Gentamicin S/R	Imipenem S/R
Pseudomonas aeroginosa	-	14/4	13/4	-	8/0	4/3
Enterobacter cloacae	-	8/0	3/0	-	1/1	3/0
Escherichia coli	0/0	2/1	20	1/1	-	1/0
Klebsiella pneumoniae	3/5	5/4	4/4	1/3	4/1	2/1
Acinetobacter baumanii	0/2	3/6	1/3	1/2	5/0	0/2
Serratia marcescens	0/1	-	3/0	1/0	2/0	-
Stenotrophomonas maltophilia	-	2/0	-	-	-	-
Multidrug-resistant *Acinetobacter baumanii*	0/1	0/2	0/1	-	-	-
Staphylococcus aureus	6/1	3/0	-	-	-	-
Methicillin-resistant *Staphylococcus aureus*	0/4	0/2	-	-	-	-
Staphylococcus coagulase	0/2	-	-	-	-	-
Streptococcus pyogenes	0/1	1/0	-	-	-	-

Regarding age, *Pseudomonas aeruginosa* was the most common bacteria in adults (55%) compared to pediatric patients (45%). *Enterobacter cloacae* occurred more often in adults (59%) compared to pediatric patients (41%). *Klebsiella pneumoniae* was more common in adults (75%) than in pediatric patients (25%) in the burn unit. *Acinetobacter baumannii* occurred more in adults (62%) compared to pediatric patients (38%). Complications after infection in burn patients are presented in Table 3.

**Table 3:** Type of complications after burn infection.

Type of complications	n
Shifted to the intensive care unit	55
Scaring	26
Sepsis	24
Readmission	20
Multiple-organ failure	3
Renal failure	4
Gastrointestinal bleeding	4
Respiratory failure and cardiac arrest	2
Organ loss	1

## Discussion

This study aimed to determine the most frequent bacterial infections among burn patients and their sensitivity and resistance patterns to initial antibiotics to help establish a protocol for antibiotic administration among burn patients in KAMC. In this study, we retrospectively collected data from 109 burn patients who developed infections in the burn ward. A total of 33 bacterial organisms were cultured from urine, stool, blood, and respiratory secretions. Out of the 109 burn patients with infections, gram-negative bacteria were the most common cause of infections, as they infected 60 cases out of the total sample size (55%). These were followed by gram-positive bacteria with nine cases out of the 109 patients (8.2%), while 39 out of 109 (35.7%) burn patients developed both gram-negative and gram-positive bacteria. Only one patient developed a concomitant gram-negative bacterial and fungal infection (0.9%). Similar findings were reported in another study performed by Zheng et al., who reported that gram-negative bacteria (54.1%) are the leading co-infectious organisms along with *Staphylococcus aureus* followed by gram-positive bacteria (36.1%) [9].

The most frequent infectious organisms in burn patients were *Pseudomonas aeruginosa*, *Enterobacter cloacae*, *Klebsiella pneumoniae*, *Acinetobacter baumannii*, and *Staphylococcus aureus*. In their study, Joy et al. reported *Acinetobacter baumannii* and *Pseudomonas aeruginosa* as the most frequent gram-negative infectious agents at 26.56% each, followed by *Klebsiella *comprising 17.19% of the cases. *Staphylococcus aureus* was the leading gram-positive bacteria found in the cultures. They also reported that *Acinetobacter* species were mostly resistant to piperacillin/tazobactam at 84.71% which is comparable to the 80% resistance found in this study. There was an 82.35% resistance to gentamicin; however, in our study, there were no resistant cases of *Acinetobacter *to gentamicin. Similarly, *Pseudomonas aeruginosa* was found to be resistant to gentamicin and piperacillin/tazobactam at 62.35% and 52.94% of the cases, respectively. In contrast to this study, we only found 20% resistant cases of *Pseudomonas aeruginosa* to piperacillin/tazobactam and there were no resistant cases to gentamicin [10]. Escandón-Vargas et al., in their prospective study, observed that gram-negative bacteria were the most common microbes in infections among burn patients and found that *Staphylococcus aureus*, *Pseudomonas*, and *Acinetobacter baumannii* are the most commonly reported organisms [11]. An extensive literature search revealed comparable results as *Pseudomonas aeruginosa*, *Acinetobacter baumannii*, *Staphylococcus aureus*, and *Klebsiella Pneumoniae* were the most frequent organisms found in burn patients with infections, along with other varying species with similar patterns of resistance and sensitivity for *Acinetobacter* and *Pseudomonas*, among others [2,5-7,10,12-21]. There was no significant pattern in organisms within the six-year period of our study.

In their recent retrospective study among 68 patients, Lopes et al. reported an average length of 28.4 days of hospital stay compared to 46.05 days in our study [22]. Mater et al. in their study on burn patients reported the average age of 19.79 years and the area affected due to burns at 16.54%. Overall, 68.4% of the patients were males and 31.6% were females. They reported that the most common organisms were MRSA and *Pseudomonas aeruginosa* [23].

Scalds were the most frequent cause of burn in our study at 39.4%, followed by flame burns at 32.1%. Gulhan et al. and Ramirez-Blanco et al. also found scalds to be the leading cause of burns in their studies, followed by flame burns [12,24]. Scalding burn wounds with long hospital stays, long-term administration of broad-spectrum antibiotics, delayed wound processing, and prolonged invasive procedures provide a favorable environment for the development of multiple-drug-resistant strains of *Pseudomonas aeruginosa*, which has been demonstrated to resist antibiotic therapy via mutations in the efflux system and outer membrane proteins, production of inactivating enzymes such as lactamase, and bacterial biofilm formation [25,26].

In KAMC, there are no specific guidelines for initial antibiotic administration to treat infections in burn patients. Antibiotic drugs were selected by doctors based on experience before identifying pathogens. Antibiotics were applied to wounds or administered systemically when they could not be applied to burn wounds. Similar guidelines or strategies are followed worldwide [27,28].

During data collection and analysis, we encountered some limitations. First, some antibiotic sensitivities were not cultured in our labs, for example, ofloxacin and silver sulfadiazine. Second, there were some damaged and missing physical patient files. Third, the sample size was very small due to the scarcity of burn patients in KAMC. Lastly, we were granted delayed access to Best Care.

## Conclusions

This study characterized the pathogens of 109 in-patients in the burn unit at KAMC between June 2016 and November 2021. Among the 109 patients, *Pseudomonas aeruginosa* was the most frequent organism that caused infections, while *Enterobacter cloacae* was the second most frequent. Understanding the distribution of bacterial infections will be crucial for the development of treatment guidelines designed to reduce hospital-acquired infections and reduce drug resistance.

In-depth attention should be paid to gram-negative bacteria, specifically *Pseudomonas aeruginosa *and* Enterobacter Cloacae*. Understanding the antibacterial resistance and sensitivity of infections in burn patients in KAMC will be crucial for the establishment of treatment guidelines to reduce hospital length of stay as well as drug resistance.
